# The Impact of Various Time Intervals on the Supragingival Plaque Dynamic Core Microbiome

**DOI:** 10.1371/journal.pone.0124631

**Published:** 2015-05-05

**Authors:** Wen-xin Jiang, Yue-jian Hu, Li Gao, Zhi-yan He, Cai-lian Zhu, Rui Ma, Zheng-wei Huang

**Affiliations:** Department of Endodontics, Ninth People’s Hospital, Shanghai Jiao Tong University School of Medicine, Shanghai Key Laboratory of Stomatology, Shanghai, China; Loyola University Chicago, UNITED STATES

## Abstract

**Objective:**

The aim of this study was to examine the influence of various time intervals on the composition of the supragingival plaque microbiome, especially the dynamic core microbiome, and to find a suitable observation interval for further studies on oral microbiota.

**Methods and Materials:**

Eight qualified volunteers whose respective age ranges from 25 to 28 years participated in the present study. The supragingival plaque was collected from the buccogingival surface of the maxillary first molar at eight time slots with different intervals (day 0, 1 day, 3 days, 1 week, 2 weeks, 3 weeks, 1 month, and 3 months). Bioinformatic analyses was performed based on 16S rDNA pyrosequencing (454 sequencing platform) targeting at the hypervariable V4–V5 region, in order to assess the diversity and variation of the supragingival plaque microbiome.

**Results:**

A total of 359,565 qualified reads for 64 samples were generated for subsequent analyses, which represents 8,452 operational taxonomic units identified at 3% dissimilarity. The dynamic core microbiome detected in the current study included five phyla, 12 genera and 13 species. At the genus level, the relative abundance of bacterial communities under the “1 day,” “1 month,” and “3 months” intervals was clustered into sub-category. At the species level, the number of overlapping species remained stable between the “1 month” and “3 months” intervals, whereas the number of dynamic core species became stable within only 1 week.

**Conclusions:**

This study emphasized the impact of different time intervals (days, weeks and months) on the composition, commonality and diversity of the supragingival microbiome. The analyses found that for various types of studies, the time interval of a month is more suitable for observing the general composition of the supragingival microbiome, and that a week is better for observing the dynamic core microbiome.

## Introduction

The oral cavity of humans is colonized by an enormous number of microorganisms that may include at least 700 species [1]. With the advent of innovative technology, especially the next generation sequencing platform, more and more bacterial phylotypes have been revealed in oral microbial communities, and the observed number of species has undergone upward adjustment [2,3]. Thus, it is not surprising that the composition and variation of oral microbiota is of profound importance for human health and physiology [4]. A better understanding of this complex micro-ecosystem is essential to guide intervention in the microflora and disease prevention [5,6].

Due to the inter- and intra-individual complexities of oral microbiota, studies on oral microbiota tend to focus on “microbial commonality,” which refers to the “core microbiome”, a fundamental issue raised by human microbiome projects. Generally, the term “core microbiome” is defined as the set of microbial operational taxonomic units (“OTUs”) that are shared among microbial assemblages associated with a particular habitat (e.g. the oral cavity), and present in all or the vast majority of humans [7,8]. An analysis of the oral microbiome in multiple sites from three adults, using 454 pyrosequencing, revealed a large overlap in species-level phylotypes and unique sequences among them, and the “shared OTUs” contributed to 94% of the sequences [3]. Another study on salivary microbiota from five individuals revealed eight genera that were shared by all individuals at all time slots, and comprised the “core taxa” [9]. Further studies of the oral microbiota confirmed the existence and general composition of the core microbiome [10,11]. It is increasingly recognized that the core microbiomes (or “core taxa”, “common taxa”, “shared OTUs”, etc.) may be critical to the ecosystem function of the community within the oral cavity, and play an important role in the maintenance of health.

The characterization and evaluation of the core microbiome require the application of ecological theory and bioinformatic analysis, and the fact that there are various definitions of core microbiome adds to its complication. As mentioned above, the most typical approach used to define a core is to find the overlap of microbial communities from different subjects based on OTU occurrences, which results in a Venn diagram [12,13]. Fundamentally speaking, the “core microbiome” is composed of shared microbial members and reflects the commonality of communities. Because of the ubiquity of the core microbiome, exploration of this population is important for understanding the stable and complex micro-ecosystem in the oral cavity. Defining the core is the first step in uncovering the healthy community, and will further establish a universal standard for the line between health and disease.

As mentioned above, the “core microbiome” is a bacterial community shared by all subjects without taking time as a factor into consideration. However, some investigators have reported that time can be a factor that influences the composition of the micro-ecosystem [8]. Thus, the term “dynamic core” was introduced in the current study to investigate the influence of various time intervals on the supragingival plaque microbiota. Dynamic core microbiota is defined as an assembly of bacteria existing at every sampling time of each individual. In other words, dynamic core could be understood as the core microbiome relating to the time series. A similar definition was employed previously by Lazarevic *et al*. [9]. They analyzed the saliva samples from five adults at three time slots across 29 days, and found that the bacterial composition was stable within at least 5 days. However, the time span and the intervals they set were still limited. For the purpose of observing the effect of various time intervals on the bacterial communities, and of finding a more stable dynamic core, time intervals set in this study ranged from days (day 0, 1 day, 2 days), weeks (1 week, 2 weeks, 3 weeks) to months (1 month, 3 months). Determining a suitable time boundary for observing the supragingival plaque microbiome is highly significant. This will guide further studies on the microbiome difference between health and disease. In addition, unnecessary extension of the observation time will increase the cost of the project, complicate the sampling process, and reduce the compliance of participants over a long time period. Extending the time will also result in missing the optimal opportunity for intervention and treatment.

## Materials and Methods

### Enrollment of subjects

The study was approved by the ethics committee of Shanghai Jiao Tong University, and written informed consent was obtained from all participants before enrollment. Eight healthy Chinese adults, including four males and four females, participated in the study. All the participants, whose respective age ranged from 25 to 28 years, were postgraduate students boarding at the College of Medicine of Shanghai Jiao Tong University for at least 1 year, eating in the school canteen and having the same oral hygiene habits (including brushing teeth twice a day). Exclusion criteria included (i) the presence of carious maxillary first molars or any untreated cavitated carious lesions and oral abscesses, (ii) periodontal disease or periodontal pockets > = 4 mm, (iii) clinically meaningful halitosis as determined by the organoleptic assessment of an experienced clinician, (iv) the use of antibiotics within 3 months before the study, (v) previous diagnosis of Sjogren’s syndrome or any disease characterized by xerostomia, (vi) inability to maintain oral hygiene during the study, (vii) the habits of smoking or drinking, and (viii) any other disease affecting oral health status or systemic health status.

### Sample collection

For each of the eight subjects, supragingival plaque samples were collected at eight time slots within 3 months, which were labeled by the time duration (day 0, 1 day, 3 days, 1 week, 2 weeks, 3 weeks, 1 month and 3 months) according to the methods mentioned in the Manual of Procedures for the Human Microbiome Project (http://hmpdacc.org/tools_protocols/tools_protocols.php). Supragingival plaque samples were obtained by a sterile Gracey curette from the buccogingival surface of maxillary first molars after the site had been isolated with cotton and dried. Repetitious scraping of the same site was necessary for obtaining a sufficient amount of specimen. The collected plaque sample was released from the curette by agitation in 300 μL TE buffer [10 mmol/L Tris-HCl (pH 7.5) and 1 mmol/L ethylene diaminetetraacetic acid]. All samples were immediately transported on ice to the laboratory for DNA extraction.

### DNA Extraction and Pyrosequencing Analysis

The samples were lysed in a Mini-Beadbeater-16 (Biospec Products, Bartlesville, OK, USA) according to the manufacturer’s instructions. The total genomic DNA was obtained from the lysate using a Bacterial Genomic DNA Extraction Kit (QIAGEN, Valencia, CA, USA). All DNA was stored at −20 °C before further analysis. Polymerase chain reaction (“PCR”) amplification of the 16S rDNA hypervariable V4–V5 region [12] was carried out using the forward primer, 515F: (5′-GTG CCA GCM GCC GCG GTA A-3′) and the reverse primer, 926R: (3′- CCG TCA ATT YYT TTR AGT TT-5′). The 454 GS FLX Adaptor A (forward) and Adaptor B (reverse) were contained in the primer. An 8-base unique barcode sequence was added to each forward primer as an identifier for sequences from the different samples. The PCR program started with an initial denaturation for 5 minutes at 95 °C, followed by 30 cycles of denaturation (30 s at 94 °C), annealing (30 s at 56 °C), extension (30 s at 72 °C) and thereafter ended with an extension (10 min at 72 °C). Emulsion PCR and pyrosequencing were performed with standard Roche 454 GS-FLX recommendations. A pyrogram was used to identify raw sequences by detecting the signal peak. Sequences that were less than 200 bp, containing ambiguous bases or homopolymeric stretches, had a low quality score (< 25), and, if identified as a chimeric artifact, were discarded after removing the primer sequences and 8-bp barcodes. The qualified sequences were submitted to the SILVA database (SILVA 106; http://www.arbsilva.de) for taxonomic analysis. MOTHUR (version 1.25.1; http://www.mothur.org/) was applied to generate the OTUs and OTU rarefaction curves. Community richness and diversity indices (ACE, Chao1, Good’s coverage, Shannon–Wiener and Simpson diversity indices) were also determined at the 3% dissimilarity by the MOTHUR program. Statistical analysis was performed by the SAS Statistical Analysis System (version 8.02; SAS Institute Inc., USA). The rarefaction curve was generated by the QIIME toolkit (http://qiime.org/) [14]. Heatmap was created in MATLAB by the clustergram process (version 7.12.0; MathWorks Inc., USA).

### Sequence Deposition

Sequences were deposited in the NCBI sequence read archive (http://www.ncbi.nlm.nih.gov/Traces/sra/) under accession number SRP049987.

## Results

### Overall sequence data

A total of 359,565 reads for 64 supragingival plaque samples were generated by high-throughput sequencing. The average number of sequences observed for each individual at one sampling time was 5618 per sample (SD = 923.8) and the mean length per read ranged from 354 to 367 bp (SD = 3.2). After blasting to the SILVA database, 8452 OTUs were identified at 3% sequence dissimilarity. The average number of observed OTUs, Shannon–Wiener, Simpson index, ACE and Chao1 richness estimators at each sampling time during the 3 months are summarized in Table 1, and represent the quantitative assessment indices of the diversity and richness of the bacterial communities. All the applicable values in Table 1 were averaged by eight subjects.

**Table 1:** Average number of observed OTUs, Shannon and Simpson diversity indices, richness estimators (ACE and Chao1) and phylotype coverage of supragingival plaque bacterial communities across different time intervals.

Time duration	OTUs	ACE	Chao 1	Shannon	Simpson	Coverage
Day 0	1165	4005	2586	5.49	0.01576	0.877
1 Day	1151	3985	2573	5.50	0.01531	0.875
3 Days	1158	4109	2609	5.51	0.01531	0.873
1 Week	1186	4177	2646	5.54	0.01464	0.872
2 Weeks	1186	4095	2607	5.56	0.01452	0.875
3 Weeks	1150	3975	2527	5.48	0.01593	0.879
1 Month	1129	3862	2467	5.46	0.01590	0.882
3 Months	1134	3824	2455	5.46	0.01563	0.885

All the applicable values were averaged by eight subjects.

**Fig 1:**
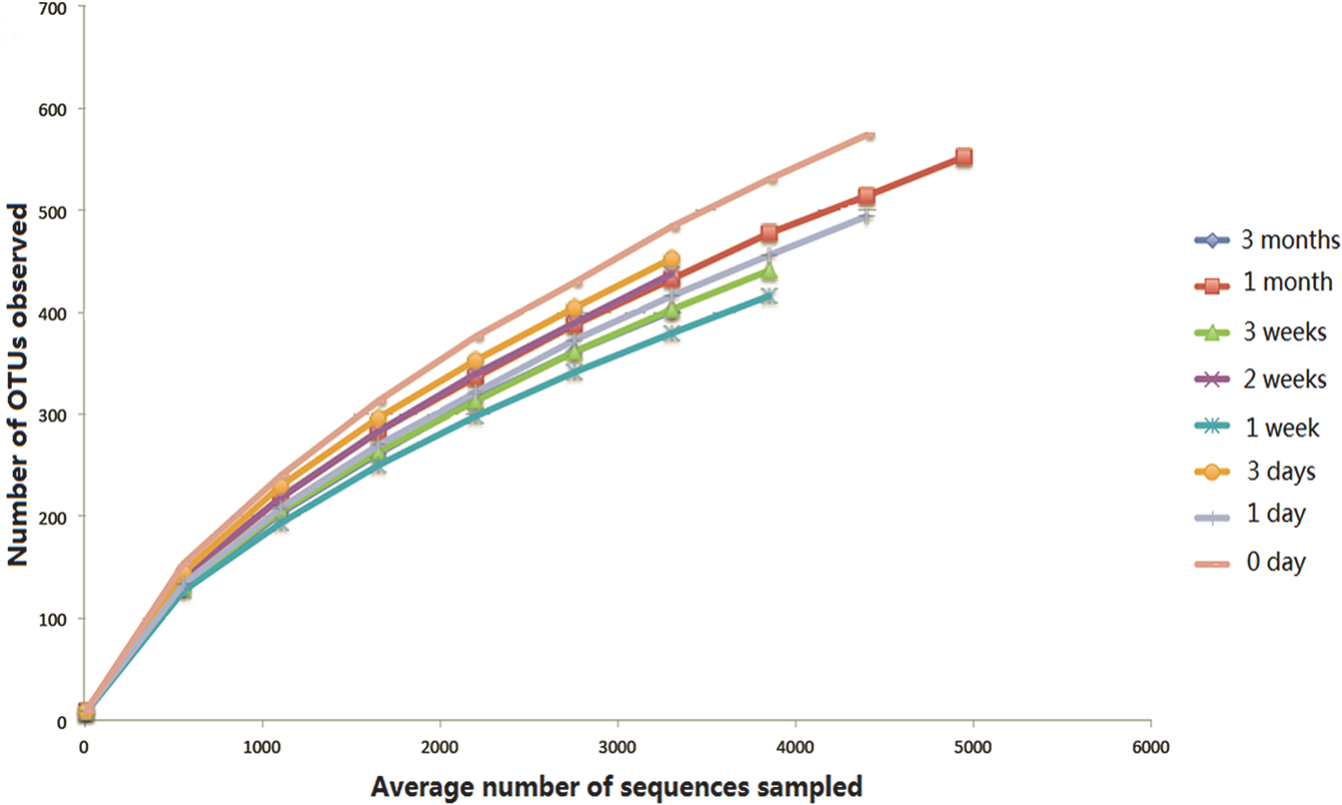
The rarefaction curves for different time intervals. The relationship between the number of OTUs observed and the average number of sequences sampled over eight sampling times (day 0, 1 day, 3 days, 1 week, 2 weeks, 3 weeks, 1 month and 3 months) are profiled by the rarefaction curves (0.03 dissimilarity level).

Rarefaction curves in Fig 1 profiled the correlation of the average number of sequences sampled and the OTUs observed across eight time intervals (day 0, 1 day, 3 days, 1 week, 2 weeks, 3 weeks, 1 month and 3 months) at a 3% cutoff (S1 Table). The 3% cutoff was generally used in the current study for species-level analysis, whereas a 5% threshold was also accepted for higher-level analysis [15]. At a 3% dissimilarity level, the average number of OTUs observed was 1008 ± 203.2, as compared to the average number of OTUs of 637 ± 133.9 obtained at 5% level.

### Richness and diversities of dynamic core microbiota

**Fig 2:**
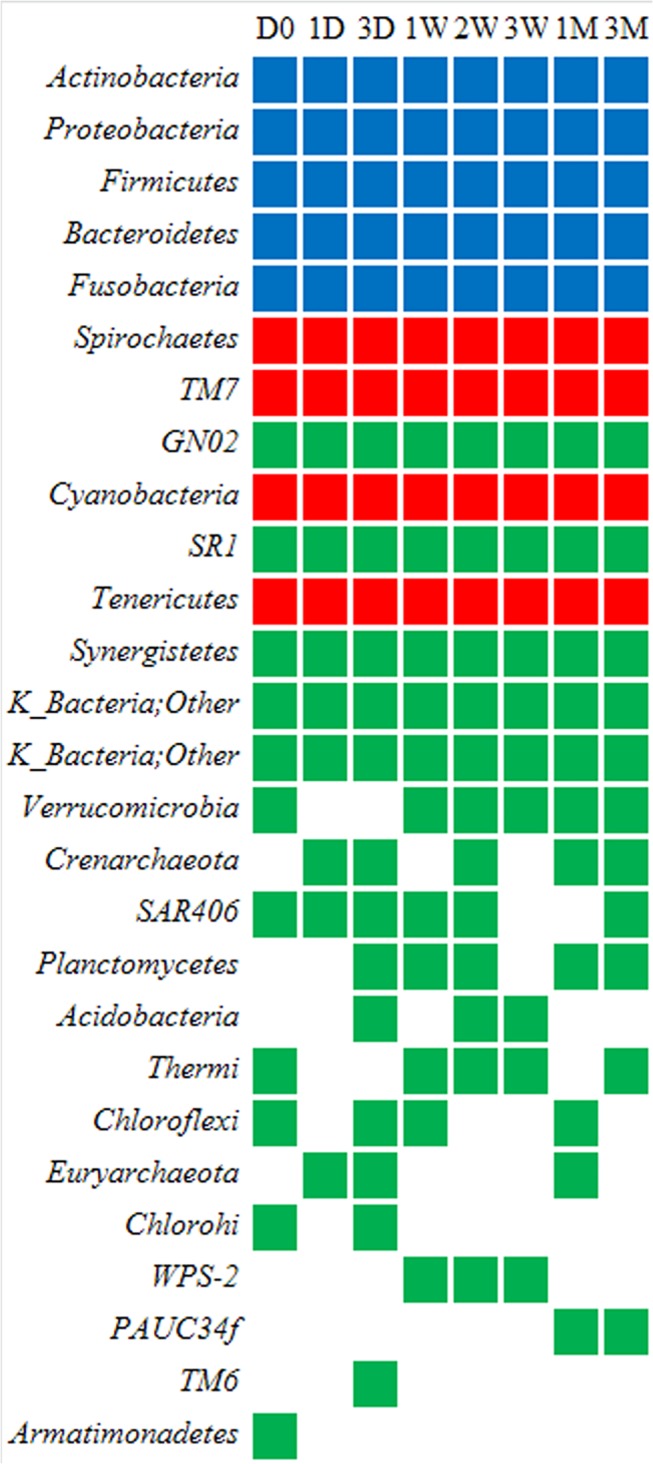
Existence of 27 phyla at each sampling time. Data given in the figure are a pool of eight subjects. 3M, 3months; 1M, 1 month; 3W, 3 weeks; 2W, 2 weeks; 1W, 1 week; 3D, 3 days; 1D, 1 day; D0, day 0. Corresponding color block would be obtained if a phylum was detected at each time slot. The five dynamic phyla core illustrated by the blue blocks are common in all the subjects across the different intervals. The red blocks are the phyla common in all subjects but not across all the time periods. The four phyla (indicated by red blocks) and the five phyla (indicated by blue blocks) that common in all the eight subjects constitute the core phyla. The other 18 phyla that are failed to be found in all subjects are marked by green blocks. They are transient phyla.

**Fig 3:**
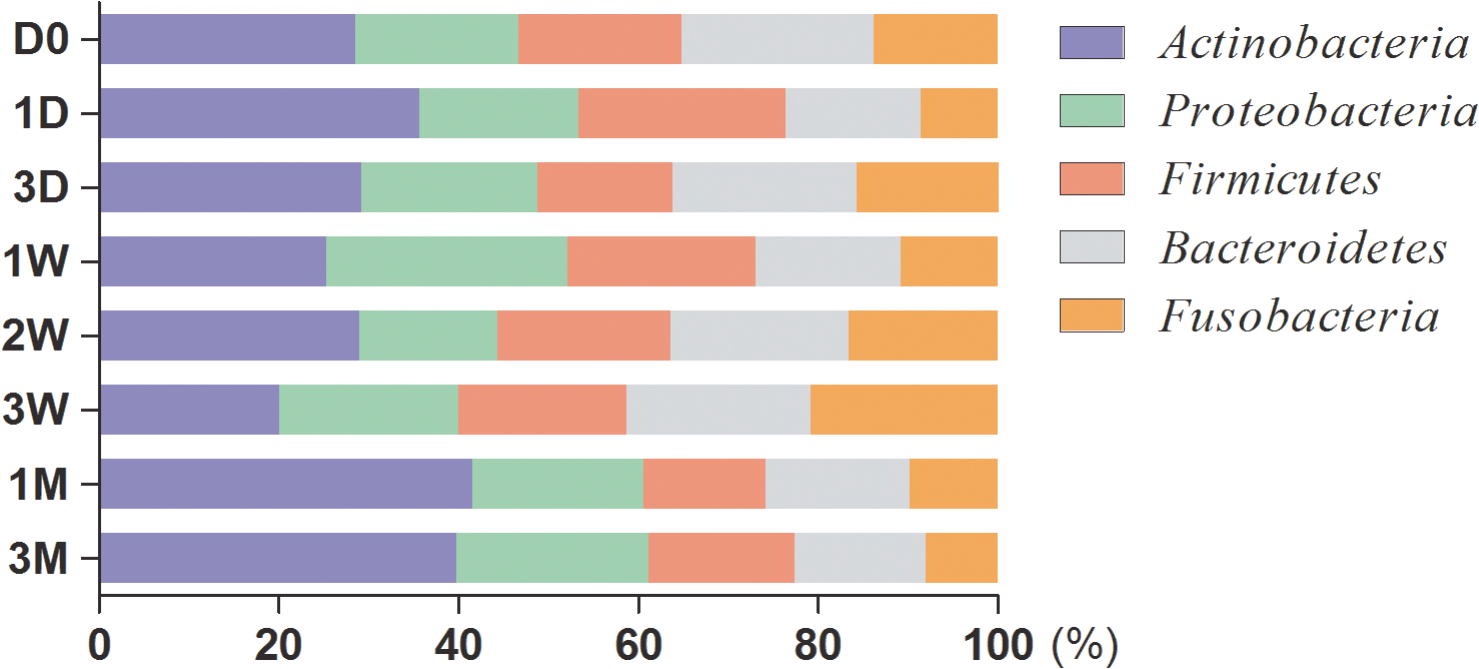
The relative abundance of dynamic core phyla. The relative abundance of five dynamic core microbiomes over eight time slots. The data calculated in the figure were scaled up to 100%. 3M, 3months; 1M, 1 month; 3W, 3 weeks; 2W, 2 weeks; 1W, 1 week; 3D, 3 days; 1D, 1 day; D0, day 0.

At the phylum level, after 16S rDNA sequencing, 359,565 reads from plaque resources were generated, identified and classified into 27 phyla. Among them, the core bacterial communities comprised nine phyla. However, only five phyla subsisted over time and qualified to be the dynamic core phyla, representing a 92.72% relative abundance. These microbiota were *Actinobacteria*, *Proteobacteria*, *Firmicutes*, *Bacteroidetes* and *Fusobacteria*. The different color blocks in Fig 2 illustrated the subsistence of the phyla across various time intervals. Briefly, if a phylum could be detected at one time slot, the corresponding color block would be obtained. The data for Fig 2 was a pool of all the eight participants. The five dynamic phyla core illustrated by the blue blocks were common in all the subjects across the different intervals. The red blocks were the phyla common in all subjects but not across all the time periods. Thus, the four phyla (indicated by red blocks) and the five phyla (indicated by blue blocks) that common in all the eight subjects constituted the core phyla. The other eighteen phyla (totally 27 phyla) that failed to be found in all subjects were marked by green blocks. They were transient phyla (S2 Table). Fig 3 demonstrated the relative abundance of the five dynamic phyla core at each time slot. To facilitate comparison, the percentage data in the figure was scaled up to 100%. *Actinobacteria* was the overwhelming phylum found in the majority of time intervals. In fact, five phyla represented an average of 92.72% of relative abundance, which was dominated by *Actinobacteria* (25.18%), and followed by *Proteobacteria* (19.42%), *Firmicutes* (17.79%), *Bacteroidetes* (17.65%), as well as *Fusobacteria* (12.69%).

**Fig 4:**
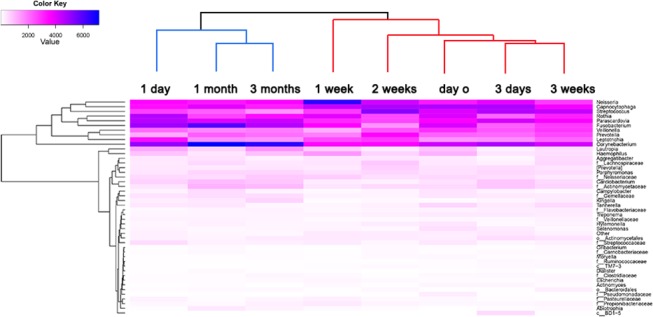
Heatmap of 48 genera across eight sampling times. The relative abundance of 48 predominant genera demonstrated by the change in color. Each column represents the number of sequences of the corresponding genus. The tree at the top was generated by the Clustergram process (Matlab) that is used to cluster the time points with similar bacterial composition.

At the genus level, after removing the unclassified sequences, 405 various genera from supragingival plaque bacterial communities were observed. Among them, 65 different genera were common in all subjects, but only 12 taxa were obtained in every time period over 3 months. This was designated as the dynamic genera core. Fig 4 was a heatmap that uses the gradual changing colors to describe the change in the relative abundance through time by genera that had a > 1% relative abundance. A clustergram process could be used to describe the similarity among the samples based on the distance. If the bacterial composition of the two time slots were very similar, they would be clustered into one sub-category. As demonstrated in Fig 4, it could be observed that among the three different levels of time interval (day, week and month), the time duration of “1 month” and “3 months” were in one sub-category (blue color) at the genus level. Referring to the heatmap in Fig 4, the 12 dynamic genera core were dominated by *Corynebacterium* (10.80%), *Capnocytophaga* (9.09%), *Neisseria* (8.67%), *Parascardovia* (8.34%), *Rothia* (8.28%), *Fusobacterium* (7.66%), *Streptococcus* (7.42%), *Leptotrichia* (4.94%), *Prevotella* (4.86%), *Veillonella* (4.82%), *Lautropia* (1.7%) and *Cardiobacterium* (1.5%), all of which were consistently part of the relative abundance (overall 78.08%) manifested by the darker colors.

### Comparison of remaining OTUs across various time intervals between overlapping species and dynamic core species

**Fig 5:**
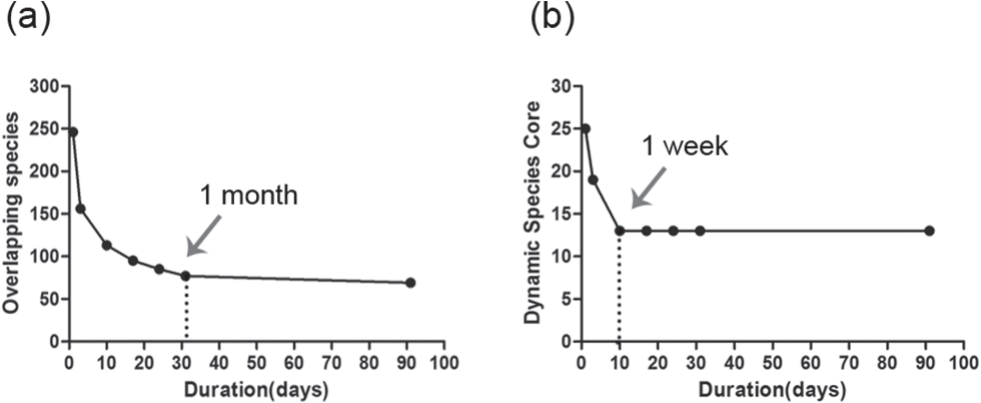
(a) The number of overlapping species observed at each time duration over 3 months. The horizontal axis represents the time duration. The number of overlapping species decreases quickly among the time interval of days and weeks. No significant differences can be observed over one month (marked by the arrow). (b) The number of dynamic core species across each time interval. The number of the dynamic core species decrease quickly for the first 3 days and become steady (13 dynamic core species) over one week (marked by the arrow).

At the species level, 8452 OTUs were found at the 0.03 cutoff. There was a dramatic decrease in the number of overlapping species as a function of time (Fig 5A). The rate of decrease was the greatest during the first 3 days. In terms of days, the number of overlapping species decreased from 246 (SD = 37.2) at day one to 156 (SD = 31.4) at day three; in terms of weeks, overlapping number of species remained at 113 (SD = 16.6) as at the end of the first week, slightly declined to 95 (SD = 13.3) as at the end of the second week and further decreased to 85 (SD = 12.2) as at the end of the third week; whereas in terms of months, overlapping number of species slightly decreased from 77 (SD = 11.6) as at the end of the first month to 69 (SD = 14.7) as at the end of the third month, representing a steady level. It is worth noting that there were statistically significant differences between any two time slots (p < 0.05), except for “subsistence for 1 month” and “subsistence for 3 months” (p > 0.05).

However, the dynamic core species (Fig 5B) were comparatively stable throughout various time intervals (days, weeks to months). Forty-six core species were detected at the first sampling time (day 0). Twenty-five core species were common at 1 day, and 19 were common at 3 days. At the 1 week time slot, the number of core species dropped to 13, and remained the same at 2 weeks, 3 weeks, 1 month and 3 months. These 13 dynamic core species, which subsisted for 1 month comprised 0.15% of all species detected, but represented a relative abundance of 47.63%. Among them, 11 species had ≥ 1% relative abundance over at least one time slot, and the other two species (*Lautropia* and *Cardiobacterium*) had a very low relative abundance (less than 0.5% relative abundance).

## Discussion

The oral cavity contains one of the most diverse microbiomes in the human body. In the oral cavity, plaque and saliva are the repositories of oral microbes, including viruses, fungi, protozoa, archaea and bacteria and these microorganism constitute a micro-ecosystem [16,17]. In the healthy state, the micro-ecosystem is balanced; but it can change dramatically in some extreme situations, including head-and-neck radiotherapy [11,18], and AIDS [19]. The change could result in numerous oral diseases, the most common ones being oral malodor [20], dental caries [21,22], gingivitis and periodontitis [23]. Moreover, systemic status, such as leukemia, has significant impact on the structure of oral microbiome as well [24]. Therefore, as a contribution to further studies, it is vital to establish a framework of oral bacterial communities for healthy populations.

In recent studies, scientists observed that the oral cavity is a highly heterogeneous ecological system containing distinct niches with significantly different microbial communities. In other words, bacterial communities in oral cavity are site-specific. Xin Xu *et al* [25] collected the saliva, supragingival dental plaque and buccal swabs from 66 healthy Han ethnicity people and observed that the largest number of OTUs can be detected in supragingival plaque (13333 OTUs). *Proteobacteria*, *Firmicutes*, *Fusobacteria*, *Bacteroidetes* were richly represented in supragingival plaque compared with that in saliva and mucosa. However, the current study focused on the various time interval as a main variable, which was likely to impact on the composition of dynamic core microbiome of supragingival plaque among healthy individuals. For the purpose of controlling other variables that might influence the observation, supragingival plaque samples were unified collected from the buccogingival surface of maxillary first molars in this study.

The “core microbiome” is considered to be the essential part for comprehending the oral microecosystem [5]. Egija Zaura *et al*. [3] obtained the first insight into the “core microbiome” among three healthy subjects by high-throughput DNA sequencing techniques, and argued that the “core microbiome” plays a vital role in maintaining a healthy status, diagnosing and treating diseases at an early and reversible stage. Consequently, much effort has been put into defining the core microbiome from different sites in the oral cavity for people of various races, ages and health statuses [16,26]. However, to our knowledge, few studies have focused on the change of the core microbiome composition over time. Thus, to avoid the interference from transient bacterial communities, observing a stable microbiome over a period of months was required. Longer observation periods would not be beneficial for a timely and effective diagnosis, intervention and cure of the disease.

The various time intervals considered in this research have established the decrease in the number of overlapping species and the number of dynamic core microbiome. (Fig 5) The overlapping species refer to the species that not only exist at the current time slots but also the previous time slots. The overlapping species calculated for the time duration of “1 day” have to be detected on both “day 0” and “1 day”; the species for “3 days” are shared by “day 0, 1 day and 3 days”. Briefly, calculating the overlapping species is an effective way to observe the subsistence of bacterial communities in the oral cavity. Different from the definition of overlapping species, the dynamic core requires that the species be shared by all subjects. Whether calculating the number of overlapping species or the dynamic core, the purpose is to disregard the transient bacteria as much as possible, and consider only the most stable and vital composition of bacteria communities [27,28]. The decline in the overlapping species was distinct across days, weeks and months. Fig 5B demonstrates that the most rapid decline was obtained between the time interval of “1 day” and “3 days.” At longer time intervals, (1 week, 2 weeks and 3 weeks) the number of overlapping species appeared stable, but significant differences could still be found. Even over the longer intervals of “1 month” and “3 months”, no significant differences were found. This implies that intervals of days and weeks are not enough to eliminate the contribution of transient bacteria to the overlapping species; a period of 1 month appeared to be more suitable.

As set forth in Fig 5B, the dynamic core microbiome demonstrated a sharp decline in terms of days, indicating a variation curve similar to that of the number of overlapping species; however, in terms of weeks and months, the number of the dynamic core species was steady. Thirteen species comprised the dynamic core species. This suggests that weeks can be selected as an observation boundary for the dynamic core species. The disparity of the observation time intervals between the overlapping species and dynamic core species might be the result of the variation of the observed objects. Overlapping species contain not only the common species, but also individual-special species. The dynamic core species is a part of the overlapping species. The composition of overlapping species can better reflect the overall composition of the oral bacterial communities, but it is influenced heavily by the sample size and individual differences. Thus, compared to the dynamic core, the overlapping species need a longer observation time interval (month) to render a more precise and stable observation of bacterial communities. By contrast, the dynamic core species shared by all individuals at all time slots constantly had a high relative abundance and was not affected appreciably by the transient bacteria. The interval of a week is sufficient to observe stable dynamic core species. Furthermore, this indicates that the dynamic core bacterial communities are an essential part of the oral bacterial microbiota, which fluctuates slightly with time, protecting the oral health from caries [29], periodontitis [30,31] and other oral diseases [32].

The composition of oral bacterial communities observed both in quantity and of relative abundance differed depending on the time interval of observation. A significant decrease in the overlapping species was found between any two sampling times, except for the 1 month and 3 month time slots (Fig 5). Thus, 1 month is a boundary for the detection of overlapping species. Most of the common species identified were transient bacteria that were not present at every time slot over 1 month. However, a comparatively stable bacterial composition could be observed in the period between 1 month and 3 months. The results obtained with relative abundance at the genera level (Fig 4; the blue cluster) also supported the contention that the bacterial communities maintained a relatively stable composition between 1 month and 3 months. The time intervals set for this study were divided into three kinds, “days”, “weeks” and “months”. In Fig 4, the shortest distance represented the highest composition of microbiota at the genus level. As set out in the cluster diagram, the microbiota in the column headed “1 month” were similar to those in the column headed “3 months” under the same sub-category, which implied that the microbiota compositions of “1 month” and “3 months” were highly similar. The figure also suggests that the time interval of “month” is more likely to have stable bacterial communities. Nonetheless, for the purpose of the dynamic core only, the interval of “week” would be a more optimal choice. It is reasonable to believe that the main microbiome, especially the dynamic core microbiome found over a 1 month period will still exist over an even longer time period [33]. The data also implied that 1 month could be considered initially as the observation time span in further studies. Excessive extension of the observation time period will complicate sampling, increase the difficulty of patient compliance, impose excessive financial burden on the investigators, and, above all, may result in a delay in disease intervention and treatment [26,33].

## Conclusions

In conclusion, this study has shown the changing composition of dental plaque bacterial communities in healthy people over different time intervals. Our study has also demonstrated the composition of the dynamic core microbiome (at the phylum, genus and species levels), which plays an important role in maintaining oral health [32,34]. It is important to select different observation intervals based on what is being emphasized. A time interval of a “week” may be more suitable to obtain a stable composition of the dynamic core microbiome, whereas a time interval of a “month” is the preferred interval for studying the overlapping species. These approaches will lead to a better understanding of the overall micro-ecosystem [35].

## Supporting Information

S1 Table Data of overall rarefaction curve. (XLSX)

S2 Table common microbiota at eight time points. (XLSX)

## References

[1] Paster BJ, Olsen I, Aas JA, Dewhirst FE (2006) The breadth of bacterial diversity in the human periodontal pocket and other oral sites. Periodontol 2000 42: 80–87. 16930307 10.1111/j.1600-0757.2006.00174.x

[2] Keijser BJ, Zaura E, Huse SM, van der Vossen JM, Schuren FH, Montijn RC, et al (2008) Pyrosequencing analysis of the oral microflora of healthy adults. J Dent Res 87: 1016–1020. 18946007 10.1177/154405910808701104

[3] Zaura E, Keijser BJ, Huse SM, Crielaard W (2009) Defining the healthy “core microbiome” of oral microbial communities. BMC Microbiol 9: 259 10.1186/1471-2180-9-259 20003481 PMC2805672

[4] He XS, Shi WY (2009) Oral microbiology: past, present and future. Int J Oral Sci 1: 47–58. 10.4248/ijos.09029 20687296 PMC2949409

[5] Segata N, Haake SK, Mannon P, Lemon KP, Waldron L, Gevers D, et al (2012) Composition of the adult digestive tract bacterial microbiome based on seven mouth surfaces, tonsils, throat and stool samples. Genome Biol 13: R42 10.1186/gb-2012-13-6-r42 22698087 PMC3446314

[6] Pushalkar S, Li X, Kurago Z, Ramanathapuram LV, Matsumura S, Fleisher KE, et al (2014) Oral microbiota and host innate immune response in bisphosphonate-related osteonecrosis of the jaw. Int J Oral Sci 6: 219–226. 10.1038/ijos.2014.46 25105817 PMC5153588

[7] Turnbaugh PJ, Ley RE, Hamady M, Fraser-Liggett CM, Knight R, Gordon JI. (2007) The human microbiome project. Nature 449: 804–810. 17943116 10.1038/nature06244 PMC3709439

[8] Huse SM, Ye Y, Zhou Y, Fodor AA (2012) A core human microbiome as viewed through 16S rRNA sequence clusters. PLoS One 7: e34242 10.1371/journal.pone.0034242 22719824 PMC3374614

[9] Lazarevic V, Whiteson K, Hernandez D, Francois P, Schrenzel J (2010) Study of inter- and intra-individual variations in the salivary microbiota. BMC Genomics 11: 523 10.1186/1471-2164-11-523 20920195 PMC2997015

[10] Ling Z, Liu X, Luo Y, Yuan L, Nelson KE, Wang Y, et al (2013) Pyrosequencing analysis of the human microbiota of healthy Chinese undergraduates. BMC Genomics 14: 390 10.1186/1471-2164-14-390 23758874 PMC3685588

[11] Hu YJ, Shao ZY, Wang Q, Jiang YT, Ma R, Tang ZS, et al (2013) Exploring the dynamic core microbiome of plaque microbiota during head-and-neck radiotherapy using pyrosequencing. PLoS One 8: e56343 10.1371/journal.pone.0056343 23437114 PMC3578878

[12] Hamady M, Knight R (2009) Microbial community profiling for human microbiome projects: Tools, techniques, and challenges. Genome Res 19: 1141–1152. 10.1101/gr.085464.108 19383763 PMC3776646

[13] Shade A, Handelsman J (2012) Beyond the Venn diagram: the hunt for a core microbiome. Environ Microbiol 14: 4–12. 10.1111/j.1462-2920.2011.02585.x 22004523

[14] Caporaso JG, Kuczynski J, Stombaugh J, Bittinger K, Bushman FD, Costello EK, et al (2010) QIIME allows analysis of high-throughput community sequencing data. Nature Methods 7: 335–336. 10.1038/nmeth.f.303 20383131 PMC3156573

[15] Conlan S, Kong HH, Segre JA (2012) Species-level analysis of DNA sequence data from the NIH Human Microbiome Project. PLoS One 7: e47075 10.1371/journal.pone.0047075 23071716 PMC3468466

[16] Jiang W, Jiang Y, Li C, Liang J (2011) Investigation of supragingival plaque microbiota in different caries status of Chinese preschool children by denaturing gradient gel electrophoresis. Microb Ecol 61: 342–352. 10.1007/s00248-010-9753-z 20927511

[17] Jiang W, Zhang J, Chen H (2013) Pyrosequencing analysis of oral microbiota in children with severe early childhood dental caries. Curr Microbiol 67: 537–542. 10.1007/s00284-013-0393-7 23743597

[18] Hu YJ, Wang Q, Jiang YT, Ma R, Xia WW, Tang ZS, et al (2013) Characterization of oral bacterial diversity of irradiated patients by high-throughput sequencing. Int J Oral Sci 5: 21–25. 10.1038/ijos.2013.15 23538641 PMC3632764

[19] Mukherjee PK, Chandra J, Retuerto M, Sikaroodi M, Brown RE, Jurevic R, et al (2014) Oral mycobiome analysis of HIV-infected patients: identification of Pichia as an antagonist of opportunistic fungi. PLoS Pathog 10: e1003996 10.1371/journal.ppat.1003996 24626467 PMC3953492

[20] Yang F, Huang S, He T, Catrenich C, Teng F, Bo C, et al (2013) Microbial basis of oral malodor development in humans. J Dent Res 92: 1106–1112. 10.1177/0022034513507065 24101743

[21] Tao Y, Zhou Y, Ouyang Y, Lin H (2013) Dynamics of oral microbial community profiling during severe early childhood caries development monitored by PCR-DGGE. Arch Oral Biol 58: 1129–1138. 10.1016/j.archoralbio.2013.04.005 23664249

[22] Yamanaka W, Takeshita T, Shibata Y, Matsuo K, Eshima N, Yokoyama T, et al (2012) Compositional stability of a salivary bacterial population against supragingival microbiota shift following periodontal therapy. PLoS One 7: e42806 10.1371/journal.pone.0042806 22916162 PMC3420916

[23] Ye Y, Carlsson G, Agholme MB, Wilson JA, Roos A, Henriques-Normark B, et al (2013) Oral bacterial community dynamics in paediatric patients with malignancies in relation to chemotherapy-related oral mucositis: a prospective study. Clin Microbiol Infect 19: E559–567. 10.1111/1469-0691.12287 23829394 PMC4413823

[24] Wang Y, Xue J, Zhou X, You M, Du Q, Yang X, et al (2014) Oral microbiota distinguishes acute lymphoblastic leukemia pediatric hosts from healthy populations. PLoS One 9: e102116 10.1371/journal.pone.0102116 25025462 PMC4099009

[25] Xu X, He J, Xue J, Wang Y, Li K, Zhang K, et al. (2014) Oral cavity contains distinct niches with dynamic microbial communities. Environ Microbiol. 10.1111/1462-2920.12502 24800728

[26] Woo PC, Lau SK, Teng JL, Tse H, Yuen KY (2008) Then and now: use of 16S rDNA gene sequencing for bacterial identification and discovery of novel bacteria in clinical microbiology laboratories. Clin Microbiol Infect 14: 908–934. 10.1111/j.1469-0691.2008.02070.x 18828852

[27] Belda-Ferre P, Alcaraz LD, Cabrera-Rubio R, Romero H, Simon-Soro A, Pignatelli M, et al (2012) The oral metagenome in health and disease. Isme j 6: 46–56. 10.1038/ismej.2011.85 21716308 PMC3246241

[28] Grice EA, Segre JA (2012) The human microbiome: our second genome. Annu Rev Genomics Hum Genet 13: 151–170. 10.1146/annurev-genom-090711-163814 22703178 PMC3518434

[29] Xin BC, Luo AH, Qin J, Paster BJ, Xu YL, Li YL, et al (2013) Microbial diversity in the oral cavity of healthy Chinese Han children. Oral Dis 19: 401–405. 10.1111/odi.12018 23034082

[30] Peterson SN, Snesrud E, Liu J, Ong AC, Kilian M, Schork NJ, et al (2013) The dental plaque microbiome in health and disease. PLoS One 8: e58487 10.1371/journal.pone.0058487 23520516 PMC3592792

[31] Wang J, Qi J, Zhao H, He S, Zhang Y, Wei S, et al (2013) Metagenomic sequencing reveals microbiota and its functional potential associated with periodontal disease. Sci Rep 3: 1843 10.1038/srep01843 23673380 PMC3654486

[32] Wade WG (2013) The oral microbiome in health and disease. Pharmacol Res 69: 137–143. 10.1016/j.phrs.2012.11.006 23201354

[33] Mignard S, Flandrois JP (2006) 16S rRNA sequencing in routine bacterial identification: a 30-month experiment. J Microbiol Methods 67: 574–581. 16859787 10.1016/j.mimet.2006.05.009

[34] Li K, Bihan M, Methe BA (2013) Analyses of the stability and core taxonomic memberships of the human microbiome. PLoS One 8: e63139 10.1371/journal.pone.0063139 23671663 PMC3646044

[35] Ursell LK, Metcalf JL, Parfrey LW, Knight R (2012) Defining the human microbiome. Nutr Rev 70 Suppl 1: S38–44. 10.1111/j.1753-4887.2012.00493.x 22861806 PMC3426293

